# Retinal Pigment Epithelium Phagocytosis and Retinal Degenerative Diseases

**DOI:** 10.14336/AD.2025.0542

**Published:** 2026-06-24

**Authors:** Yuxiang Du, Yong Xia

**Affiliations:** ^1^Precision Medicine Laboratory for Chronic Non-communicable Diseases of Shandong Province, Institute of Precision Medicine, Jining Medical University, Jining, Shandong, China.; ^2^College of Pharmaceutical Engineering, Institute of Precision Medicine, Jining Medical University, Jining, Shandong, China.

**Keywords:** Phagocytosis, retinal pigment epithelial, cell signaling, retinal degeneration

## Abstract

In the mammalian retina, photoreceptors rely on the continuous renewal of their outer segments to preserve their function as light-sensing cells, thereby ensuring lifelong vision. This process entails the routine phagocytosis of shed photoreceptor outer segments (POS) by the retinal pigment epithelium (RPE). Phagocytosis of POS by RPE is a highly circadian-regulated process that is dependent on intricate and tightly controlled cellular signaling pathways. This article provides a systematic review of the research on the regulation and functional implications of the RPE phagocytic signaling system. Therefore, a deeper understanding of the role of retinal pigment epithelial phagocytosis in the pathogenesis of retinal degeneration can inform the development of potential therapeutic targets to prevent the irreversible loss of retinal pigment epithelium and photoreceptor cells, thereby preventing retinal degenerative diseases. This article summarizes the soluble and membrane-bound molecules produced by the RPE that are associated with phagocytosis and discusses their specific roles in POS phagocytosis and retinal degenerative disorders, potentially aiding in the prevention or treatment of retinal degenerative diseases.

## 1. Introduction

The retinal pigment epithelium (RPE), a highly specialized epithelial layer, maintains bidirectional interactions with photoreceptors apically and Bruch's membrane/choriocapillaris basally [1]. In the mammalian retina, individual RPE cells support approximately 30 photoreceptors while processing thousands of outer segment discs daily [2, 3]. As one of the body's most metabolically active phagocytes, RPE cells continuously clear photoreceptor outer segment fragments (POS) through a light-triggered, circadian-regulated process. Research demonstrates that sudden light onset triggers both massive shedding of POS fragments [4, 5] and induces polarized mitochondrial translocation in RPE cells, with basal-to-apical mitochondrial redistribution enhancing interactions with POS-containing phagosomes [6].

Previously reported that maximal POS clearance by RPE occurs at 1.5-2 hours after light onset [7, 8]. Recent advances in adaptive optics optical coherence tomography have revealed that peak cone phagocytosis in humans occurs within 1 hour after light onset [9]. As core circadian regulators, melatonin and dopamine coordinately orchestrate retinal diurnal rhythms by synergistically modulating both disc shedding and POS phagocytic cycles, serving as dark-phase and light-phase signaling molecules, respectively [10]. Dopamine synthesis inhibition in the early light phase significantly reduced both disk shedding and phagocytosis [11], whereas dopamine receptor knockout mice failed to exhibit the phagocytic burst after light onset [12]. In addition to visible light stimulation, RPE phagocytosis can be modulated by different environmental or molecular regulators, such as cytokines [13], insulin [14], metal ion homeostasis [15], and starvation [16, 17].

The RPE phagocytoses and degrades POS, recycling their molecular components for reuse in the visual cycle [18]. Disruption of this process can cause vision loss [19], as impaired RPE phagocytosis leads to the accumulation of undigested POS material, ultimately resulting in photoreceptor degeneration and vision impairment [20]. Notably, RPE phagocytosis stimulates local insulin production, which enhances retinal glucose uptake and metabolic homeostasis through insulin receptor (InsR) phosphorylation and subsequent upregulation of glucose transporter 4 (GLUT4). In mouse RPE, both InsR phosphorylation and GLUT4 expression exhibit circadian rhythms synchronized with phagocytic activity, peaking one hour after light onset before gradually decreasing. Importantly, genetic ablation of phagocytic receptors significantly reduces Ins2 (insulin-encoding) expression in RPE cells [16]. Dysregulated phagocytosis has been described in retinal degenerative diseases, including acquired age-related macular degeneration (AMD) [21] and hereditary retinal degenerative diseases, such as retinitis pigmentosa (RP) [22]. These diseases cause vision loss in millions worldwide; however, no definitive treatment currently exists for AMD and RP patients with photoreceptor degeneration [23].

**Figure 1.**
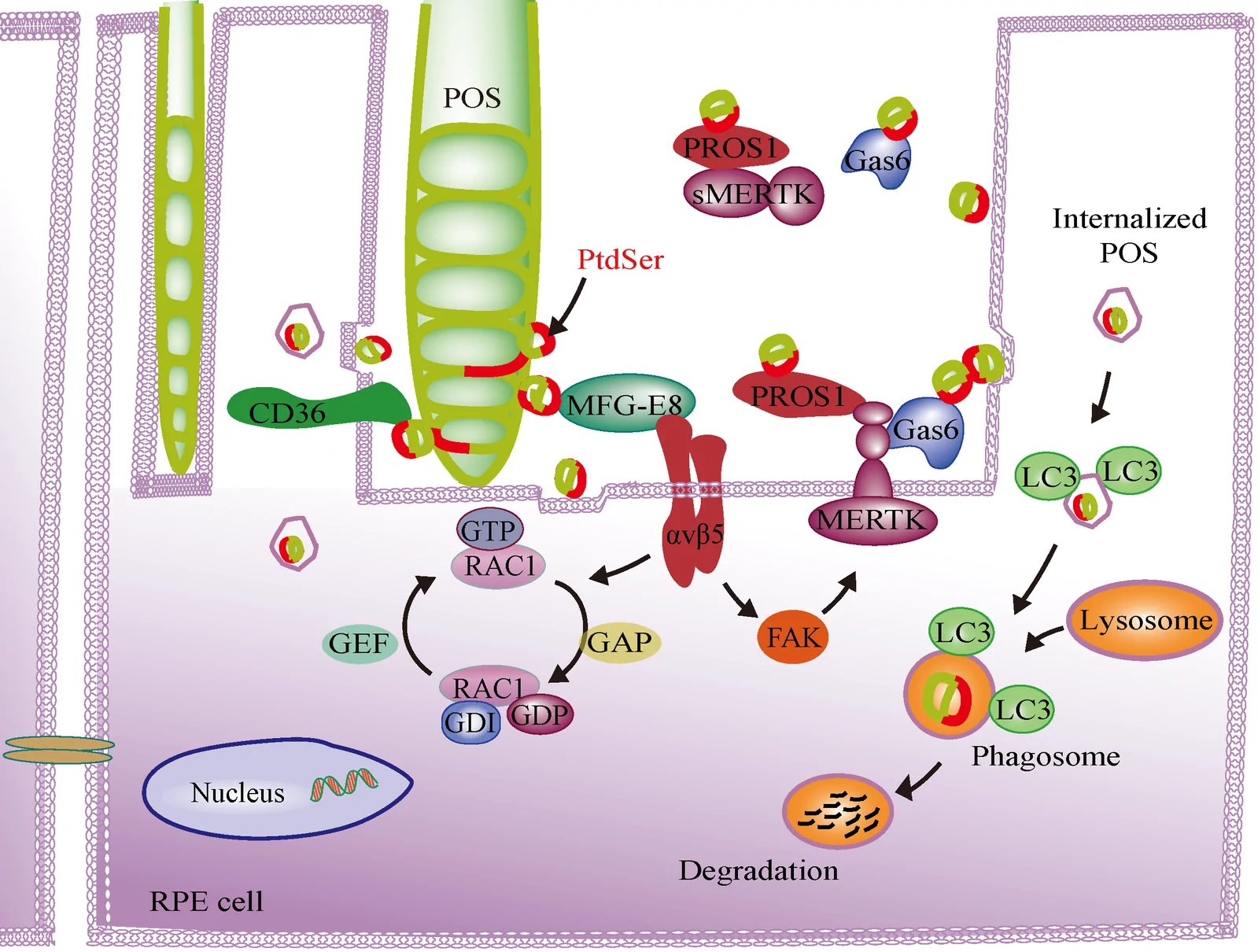
**The core mechanism of phagocytosis in retinal pigment epithelial cells**. POS shed from photoreceptor cells exhibit externalized PtdSer, enabling their binding to CD36. The αvβ5 integrin receptor engages POS via MFG-E8 bridging, triggering FAK-MERTK and RAC1-GTPase activation to recruit actin to the phagocytic cup. MERTK can also be activated through GAS6 and/or PROS1 bound to POS. Soluble MERTK (sMERTK) acts as a decoy receptor for MERTK ligands, suppressing phagocytosis. POS degradation occurs through LAP, a noncanonical autophagic process where engulfed POS are incorporated into phagosomes, followed by LC3 lipidation and subsequent lysosomal fusion to form phagolysosomes for POS cargo degradation. POS: photoreceptor outer segments; PtdSer: phosphatidylserine; PROS1: Protein S; Gas6: growth arrest-specific gene 6; MERTK: Mer receptor tyrosine kinase; sMERTK: soluble form Mer receptor tyrosine kinase; CD36: cluster of differentiation 36; MFG-E8: milk fat globule-epidermal growth factor 8; αvβ5: αv and β5 integrin; FAK: focal adhesion kinase; GTP: guanosine triphosphate; RAC1: Ras-related C3 botulinum toxin substrate 1; GAP: GTPase-activating protein; GDI: GDP dissociation inhibitor; GEF: guanine nucleotide exchange factor; GDP: guanosine diphosphate; LC3: microtubule-associated proteins 1A/1B light chain 3; LAP: LC3-associated phagocytosis; RPE: retinal pigment epithelium.

The phagocytosis of POS by RPE cells is a complex and tightly regulated process, yet the complete molecular mechanism underlying this process remains to be fully elucidated [24]. The process can be divided into three stages: recognition, ingestion, and digestion, which involve numerous regulatory genes. Key participants include αv and β5 integrin (αvβ5) [25], Mer proto-oncogene tyrosine kinase (MERTK) [26], cluster of differentiation 36 (CD36) [26], focal adhesion kinase (FAK) [27], milk fat globule-epidermal growth factor 8 (MFG-E8) [28], growth arrest-specific gene 6 (Gas6) [29], Protein S (PROS1) [30], and microtubule-associated proteins 1A/1B light chain 3 (LC3) [31] (Fig. 1). RPE cell phagocytosis dysfunction serves as a primary pathogenic trigger for retinal degenerative disorders [22]. Therefore, elucidating the molecular mechanisms through which RPE cells regulate phagocytosis could pave the way for developing effective therapeutic strategies to target retinal degeneration. This review summarizes the soluble and membrane-bound molecules produced by RPE associated with phagocytosis and discusses their specific roles in POS phagocytosis and retinal degenerative disorders. By synthesizing the current knowledge on RPE phagocytic mechanisms in degenerative retinal diseases, this study seeks to establish conceptual frameworks for innovative preventive and treatment approaches.

## 2. Retinal degenerative diseases

### 2.1 AMD

AMD, a predominant cause of global blindness among the elderly [32], currently affects approximately 196 million individuals worldwide, with projections suggesting that this number will rise to 288 million by 2040 [33]. AMD is present clinically in two distinct forms: dry (non-neovascular) and wet (neovascular). The dry subtype, representing approximately 90% of AMD cases, predominantly involves pathological alterations in the RPE and Bruch's membrane, with characteristic features including RPE atrophy and photoreceptor degeneration [34]. Wet AMD is defined by abnormal choroidal neovascularization [35]. Currently, therapeutic strategies for AMD primarily focus on managing late-stage wet AMD, while lacking effective interventions to prevent irreversible apoptosis and degeneration of RPE and photoreceptor cells [36, 37].

Studies have demonstrated that RPE is the primary site for pathological changes in AMD [38]. RPE cells in AMD exhibit multiple pathological features, including deficient POS clearance, abnormal complement pathway activation, progressive accumulation of basal deposits, and significantly increased mitochondrial oxidative stress [39]. Inana et al. demonstrated an age-related decline in RPE phagocytic activity [21]. Comparative studies reveal markedly impaired phagocytic function in RPE cells from AMD patients compared to healthy age-matched controls. Following specific binding processes, the outer segments of photoreceptors fuse with lysosomes to efficiently recycle vitamin A derivatives and lipids [21]. Impairment of this fusion process leads to lysosomal enlargement, a well-documented feature of AMD pathogenesis [38].

Growing evidence indicates that dysregulated phagocytic function in RPE cells contributes to the pathophysiology of AMD [40-42]. Oxidative stress, recognized as a major AMD risk factor, activates AMP-activated protein kinase (AMPK) while simultaneously inhibiting phagocytic activity [43]. Experimental studies have found that AMPKα2 knockdown not only decreases POS phagocytosis but also enhances cellular resistance to oxidative damage in human RPE cells [43]. This observed decrease in POS phagocytic capacity under oxidative stress conditions may reflect an adaptive protective mechanism employed by RPE cells [43]. Therapeutic approaches targeting RPE cell phagocytosis have been explored as potential treatment options for AMD [41].

### 2.2 RP

RP represents the largest class of inherited retinal disorders, marked by progressive photoreceptor cell and RPE degeneration, with no universally effective treatment currently available. Epidemiological studies report that RP affects between 1 in 3,000 and 1 in 7,000 individuals worldwide [44, 45]. Patients affected by this disorder typically manifest initial symptoms of nyctalopia, which subsequently advances to peripheral visual field constriction, progressively deteriorating to tunnel vision before culminating in total vision loss [46]. Generally, early onset RP subtypes tend to progress rapidly, often commencing around the age of 10 years, and by the age of 40-50, visual impairment becomes pronounced and refractory to treatment [45]. The characteristic fundus features of typical RP include RPE mottling, optic disc pallor, narrowed retinal vasculature, and characteristic peripheral bone spicule pigmentary deposits [46]. Although RP was previously considered incurable, advances in gene therapy now offer promising interventions that may decelerate photoreceptor degeneration and potentially recover partial visual capacity [45].

With advancements in molecular diagnostics, over a hundred pathogenic genes involved in diverse biological pathways underlying RP have been identified, including MERTK [22]. MERTK encodes the c-mer protooncogene receptor tyrosine kinase, which is essential for POS phagocytosis by RPE cells. The Royal College of Surgeons (RCS) rat model harbors a MERTK mutation that disrupts this receptor tyrosine kinase function, consequently impairing RPE phagocytic capacity [47]. Defects in MERTK impair the capacity of the RPE to phagocytose POS, thereby causing progressive degeneration of both rod and cone photoreceptors, clinically manifested as RP [48]. In humans, MERTK mutations cause RPE phagocytic dysfunction, leading to the accumulation of outer segment fragments and membranous debris, which ultimately causes severe rod-cone dystrophy with significant macular involvement [48, 49]. Notably, patients with MERTK mutations exhibit a more rapid decline in vision than those with other forms of RP [50]. Emerging evidence further links RP progression with impaired glycolysis [51, 52]. Photoreceptors receive glucose through RPE-mediated transport via surface GLUT1 transporters [53]. Studies have demonstrated that MERTK-mediated recognition of phosphatidylserine on POS outer segments induces protein kinase B (AKT) phosphorylation in RPE cells. The resulting phospho-AKT upregulates apical GLUT1 expression in RPE, enhancing glucose flux to photoreceptors. Notably, under phagocytosis-deficient conditions, reduced apical GLUT1 expression in RPE leads to intracellular glucose sequestration and photoreceptor starvation, accelerating cone degeneration in RP mouse models [52].

## 3. RPE phagocytosis of POS

Phagocytosis is a critical cellular process for the internalization and clearance of particles larger than 0.5 μm in diameter, serving vital functions in maintaining tissue homeostasis and mediating innate immunity [54]. As a receptor-mediated mechanism, phagocytosis involves three primary stages: recognition, ingestion, and digestion, culminating in the efficient disposal and elimination of particles [55]. Phagocytosis is observed in various cell types, including RPE cells, which are specialized phagocytes that play a crucial role in POS renewal [56]. POS, which are located at the photoreceptor terminals, consist of stacked phospholipid bilayers that undergo circadian-regulated shedding. Subsequently, the apical surfaces of RPE cells bind to, recognize, and internalize the shed POS, thereby facilitating the efficient clearance of photooxidative byproducts generated during phototransduction [57].

Research on the rhythmic patterns of POS phagocytosis using diverse animal models has been conducted since the late 1960s [2, 7]. Comparative studies have revealed distinct species-dependent characteristics of phagocytic rhythms. Murine models demonstrate dual peaks coinciding with both light-on and light-off transitions, indicative of robust circadian control [58], whereas feline models exhibit monophasic peaks exclusively following light onset [59]. Rodent studies in rats have documented combined light-entrained and circadian-regulated clearance patterns [60], whereas amphibian models (frogs) display strictly light-triggered phagocytosis without circadian influence [61]. Avian research in chickens has revealed biphasic light-dark transition responses [62], in contrast to zebrafish, which show light-dependent but circadian-independent clearance mechanisms [63].

Although knowledge related to RPE phagocytosis remains incomplete, significant progress has recently been made. Studies have identified critical events in POS internalization, including light-induced exposure of phosphatidylserine (PtdSer) at POS tips, which serves as an 'eat-me' signal for RPE surface receptors [27, 64]. Many receptors participate in direct PtdSer binding or indirect bridging mechanisms. POS shed from photoreceptor cells exhibit externalized PtdSer, enabling their binding to CD36 on the apical membrane of RPE cells [65]. Furthermore, the αvβ5 integrin receptor engages POS by bridging the MFG-E8 protein secreted by RPE cells. Engagement of POS with the αvβ5 integrin receptor activates FAK-MERTK [27] and Ras-related C3 botulinum toxin substrate 1 (RAC1)-GTPase [66], leading to actin recruitment to the phagocytic cup. Additionally, MERTK can be activated upon binding to GAS6 and/or Protein S in a complex with POS [67, 68]. Activated MERTK triggers MRCKβ signaling and drives cup closure and particle internalization [69]. Lysosomal degradation proceeds via LC3-associated phagocytosis (LAP), where microtubule-associated proteins 1A/1B light chain 3 (MAP1LC3/LC3) direct phagosome-lysosome fusion [57, 70, 71]. MERTK coordinates this process by remodeling the actin cytoskeleton to facilitate both substrate engulfment and LAP complex assembly [67, 72, 73]. Notably, lysosomal LAP contributes to both POS clearance and the visual cycle through 11-cis retinal regeneration [31].

Phagocytic dysfunction has significant consequences for retinal tissues, causing functional impairment and retinal degeneration [38, 41]. For instance, deficiency or mutation of αvβ5 [74] or MERTK [48] accelerates RPE degeneration. Interestingly, POS phagocytosis may upregulate peroxisome proliferator-activated receptor γ coactivator-1α (PGC-1α) through the αvβ5 integrin/FAK/PGC-1α pathway, potentially counteracting reactive oxygen species (ROS)-induced senescence [27]. In AMD, impaired LAP and excessive mitochondrial ROS drive RPE degeneration [75, 76]. Enhancing RPE phagocytic activity holds therapeutic potential for retinal degenerative diseases, either alone or as a combination therapy, although target safety and efficacy require further investigation [77, 78].

### 3.1 CD36

CD36 belongs to the B2 receptor of the scavenger receptor class B family and is a highly glycosylated secondary transmembrane protein [79]. It comprises two transmembrane domains, a large extracellular region containing ligand-binding sites, and short intracellular tails at the N- and C-termini [79]. CD36, first discovered in platelets, is widely expressed in various cell types and plays an important role in phagocytosis [80]. In the mammalian retina, CD36 is mainly expressed in RPE, microvascular endothelial cells, ganglion cells, photoreceptors, and Müller cells [79, 81, 82]. Interestingly, CD36 colocalizes with POS, consistent with its established role in RPE-mediated POS clearance [82]. Both cultured human [83] and rat [84] RPE cells express CD36, which enables their participation in POS phagocytosis through direct binding to PtdSer and phosphatidylinositol on RPE apical surfaces [83, 85]. Sparrow et al. demonstrated that the CD36 protein is not expressed by RPE cells of RCS rats, a strain characterized by hereditary retinal degeneration with RPE that is unable to phagocytose POS [86].

It has been shown that CD36 does not participate in the αvβ5 integrin-dependent phase of RPE phagocytosis but may participate in the internalization of POS [27]. Further investigations have revealed that siRNAs and antibodies against CD36 only suppress POS internalization [27]. Studies have shown that CD36 ligation at the RPE surface can partially substitute for the soluble factors necessary for the internalization of outer segments at the apical surface of the RPE. CD36 seems to play a regulatory role in modulating the rate at which RPE cells internalize phagocytosed POS [65]. CD36 expression is altered in the RPE of RCS rats devoid of MERTK, which do not internalize POS [86]. CD36 mutations cause murine retinal degeneration [81]. *In vivo*, CD36 exhibits circadian expression patterns synchronized with phagocytic peaks [87] and demonstrates enhanced activity under oxidative stress conditions resembling AMD pathology [88]. Mechanistic studies have revealed CD36's involvement in oxidized phospholipid recognition during POS internalization [89] and its coordination with Toll-like receptor 4 (TLR4) to induce metabolic changes, calcium signaling, and ROS production at POS-RPE interfaces [90]. These results suggest a direct regulatory role for CD36 in the kinetics of POS phagocytosis [87].

In addition to the direct regulation of POS phagocytosis, CD36 mediates RPE-derived microparticle (RMP) uptake, which reduces POS phagocytic capacity, promotes cellular senescence, and accelerates age-related pathological changes [91]. The receptor also influences age-dependent retinal deposit formation, with CD36 activation preventing Bruch's membrane thickening while maintaining visual function [92]. Furthermore, CD36 deficiency in rodent models precipitates progressive photoreceptor degeneration and choroidal atrophy through cyclooxygenase 2 (COX2)/vascular endothelial growth factor (VEGF) downregulation, mirroring dry AMD pathogenesis [81]. Additionally, the ability of CD36 to mediate anti-angiogenesis of Thrombospondin-1 (TSP-1) suggests that its dysfunction may contribute to neovascularization [93].

### 3.2 αvβ5 integrin

Integrins, transmembrane proteins essential for cellular adhesion and migration, regulate diverse physiological and pathological processes [94]. RPE cells express several integrin receptors [95], most of which exhibit basolateral polarization, except for αvβ5 integrin, which is uniquely localized to the apical plasma membrane [96, 97]. Moreover, the apical polarity of αvβ5 integrin is maintained autonomously by RPE cells, independent of the neural retina, and can be maintained in the RPE in culture [96]. Several independent studies have demonstrated that αvβ5 integrin is critical for POS recognition and binding during phagocytosis, as demonstrated in stem cell-derived RPE, primary cultures, and cell lines [74, 97]. *In vitro*, functional blockade of αvβ5 via antibodies or RGD peptides reduces POS binding by 84% without affecting internalization [98]. Because integrin β5 dimerizes only with the integrin αv subunit, β5 integrin knockout mice offer an opportunity to study RPE cells that are permanently and completely devoid of the αvβ5 receptor [99]. Notably, apical αvβ5 expression coincides with the onset of daily POS phagocytosis in mature rat RPE cells [98]. Further studies in mice have shown that αvβ5 integrin deficiency leads to lipofuscin accumulation, vision loss, and age-related blindness, highlighting the importance of αvβ5 in maintaining photoreceptors [96].

In the mammalian retina, the integrin ligand MFG-E8, rather than vitronectin [22], binds to the exposed PtdSer on POS, bridging them to RPE αvβ5 integrin [100]. In addition, αvβ5 integrin and MFG-E8 in RPE can regulate PtdSer exposure at the tips of the photoreceptor cell POS [101]. MFG-E8-opsonized POS engage αvβ5, triggering two pathways: FAK-dependent MERTK activation [102] and RAC1-mediated F-actin recruitment to phagocytic cups [66]. *In vivo* studies demonstrate that RAC1 activation coincides with light-initiated outer segment shedding, yet this response is abolished in αvβ5 integrin-deficient mice [66]. Additionally, MERTK-dependent feedback mechanisms can limit phagocytic particle binding of POS to αvβ5 integrin receptors on RPE cells [103].

Furthermore, the activity of αvβ5 integrin in RPE cells is precisely modulated by multiple regulatory factors. CD81 partially colocalizes with αvβ5 at the apical phagocytic surface and functions as a coreceptor to promote POS binding by forming complexes with the αvβ5 integrin [3]. Overexpression of CD81 elevates the surface levels of αvβ5 receptors and enhances the POS binding capacity in RPE cells [3]. Annexin A5 interacts with αvβ5 through its C-terminal domain, increasing receptor availability at the phagocytic surface and facilitating POS recognition [104]. Protein kinase C (PKC) activation is essential for particle binding to αvβ5, as pharmacological PKC inhibition markedly reduces αvβ5 binding activity [105]. Disruption of RPE polarity downregulates αvβ5 expression and alters its distribution from apical localization to random dispersion, thereby impairing POS phagocytosis [106]. Importantly, POS binding activates the αvβ5/FAK pathway and upregulates PGC-1α, which collectively reduces ROS generation and provides cytoprotective effects in RPE cells [27]. These regulatory mechanisms demonstrate that αvβ5 integrin mediates both retinal adhesion and POS binding during RPE phagocytosis, which are crucial for maintaining photoreceptor function and survival.

### 3.3 FAK

FAK, a ubiquitously expressed non-receptor cytoplasmic tyrosine kinase and scaffold protein, plays diverse roles in RPE function [107]. In polarized RPE cells, apically localized FAK participates in POS phagocytosis by linking integrin-mediated binding and engulfment mechanisms [108]. The activation of αvβ5 integrin induces FAK phosphorylation and mobilization, where FAK signaling is essential for POS internalization but not for initial binding [108]. FAK signaling operates upstream of MERTK phosphorylation [108, 109], with POS binding to αvβ5 receptors triggering FAK activation, which subsequently promotes MERTK relocalization and activation [102, 108], ultimately facilitating POS uptake. The coordinated activation of FAK and MERTK following POS binding represents a critical regulatory point for efficient phagocytosis [108, 110].

Research by Law et al. [111] identified Annexin A2 as crucial for the rhythmic activation of FAK during POS phagocytosis. Furthermore, dopamine 2 receptor signaling modulates daily phagocytic bursts in mouse RPE through FAK-dependent mechanisms [12]. Mitochondrial dysfunction has been shown to impair RPE phagocytic capacity [112], while oxidative stress reduces RPE phagocytic activity. Notably, the antioxidant N-acetylcysteine enhances phagocytosis by increasing phosphorylated FAK and MERTK levels [113]. Sublethal oxidative stress induced by hydrogen peroxide (H_2_O_2_) significantly inhibits POS binding and internalization by disrupting FAK and MERTK signaling, with pretreatment experiments in ARPE-19 cells demonstrating nearly complete inhibition of POS-induced FAK and MERTK activation [110]. Additionally, POS-triggered αvβ5 integrin and FAK activation upregulate PGC-1α expression and reduce senescence markers in oxidatively stressed ARPE-19 cells [27]. These findings imply that targeting the αvβ5 integrin/FAK/PGC-1α signaling axis may represent a potential therapeutic strategy for age-related retinal pathologies [27]. Beyond phagocytosis, FAK signaling influences diverse cellular processes, including survival [114], cell migration [115], cell adhesion [116], collagen gel contraction [117], and angiogenesis [118], thus playing important roles in eye diseases such as retinal ischemic-reperfusion injury [119], diabetic retinopathy [115], and neovascular age-related macular degeneration [118].

### 3.4 MERTK

MERTK belongs to the TAM (Tyro3, Axl, and MERTK) family of receptor tyrosine kinases (RTKs) and encodes a transmembrane protein with two fibronectin type III domains, two immunoglobulin-like domains, and one tyrosine kinase domain [120]. Within the RPE, only MERTK and Tyro3 are expressed among TAM RTKs [121], and MERTK is the most critical RTK for phagocytosis in RPE cells, whereas Tyro3 deficiency shows no retinal degeneration phenotype [122]. Although neither MERTK nor its upstream regulator FAK is required for initial POS binding to RPE apical surfaces, αvβ5 integrin receptors can sequentially activate these molecules to facilitate phagosome engulfment and internalization [109]. The RPE-secreted ligands GAS6 and PROS1 bridge PtdSer on POS surfaces with MERTK on RPE cells [22]. Ligand binding (Gas6 or PROS1) induces MERTK autophosphorylation, triggering downstream signaling cascades that mediate POS phagocytosis [123]. Interestingly, Gas6 and PROS1 exhibit opposing retinal functions, with Gas6 acting as an inhibitor and Protein S as a phagocytosis stimulator [30]. ADAM (a disintegrin and metalloproteinase) family proteases cleave MERTK's extracellular domain to generate soluble sMERTK [124], which functions as a decoy receptor to inhibit MERTK activation and suppress phagocytosis [125]. Studies have demonstrated that sMERTK reduces POS-RPE binding, while inhibiting MERTK cleavage enhances this interaction [30]. The rhythmic release of sMerTK may help regulate the phagocytic peak duration [30]. The balance between full-length and soluble forms of MERTK may represent a critical regulatory point for POS phagocytosis [125]. Hyperglycemia-induced ADAM9 upregulation via miR-126 downregulation impairs MERTK expression/activation, inhibiting RPE phagocytosis [125, 126]. Notably, Gas6 modestly increases the release of sMerTK, whereas Protein S markedly decreases it [30]. Therapeutic Gas6 nanoparticles sustain protein release, enhancing gene therapy efficacy in MERTK-associated RP [127].

MERTK's crucial role in POS internalization is evidenced by RCS rat-derived MERTK-deficient RPE cells that bind but fail to phagocytose POS [128]. MERTK-cleavage-resistant mice show apical phagolysosome accumulation, indicating impaired POS degradation [129]. MERTK expression can rescue the RCS phenotype, supporting its involvement in RPE phagocytosis [110]. Furthermore, MERTK mutations have been implicated in autosomal recessive RP [130]. Biswas and colleagues' research demonstrates that iPSC-derived RPE cells from the proband and parents harboring splice site and deletion variants do not affect cellular viability or morphology *in vitro*, but lead to the loss of MERTK protein and a decrease in phagocytosis, suggesting that *in vivo*, these mutations impact the RPE's ability to phagocytose POS, contributing to retinal degeneration [131]. Additional evidence comes from reports of MERTK deletions ranging from small nucleotide changes to large 91 kb segments in RP patients [132]. Karl et al. found that Gas6-mediated phagocytosis requires L-type Ca^2+^ channel activation downstream of αvβ5 integrin/MERTK signaling, indicating convergence of phagocytic signaling pathways [29]. Furthermore, recent findings have shown that acute inhibition of Rho-associated protein kinases (ROCKs) can induce POS phagocytosis in MERTK-deficient RPE cells, implying that modulating downstream signaling effectors may circumvent the need for MERTK receptor activity and restore phagocytic function [133].

### 3.5 RAC1

Rac1, a ubiquitously expressed GTPase, is a well-studied and highly conserved member of the Rho family of small GTPases. Rac1 functions as a molecular switch that cycles between its active GTP-bound and inactive GDP-bound states. Its activity is tightly regulated by guanine nucleotide exchange factors (GEFs) that promote Rac1 activation and localization to the plasma membrane, GTPase-activating proteins (GAPs) that favor the on/off (GTP/GDP) switch, and GDP dissociation inhibitors (GDIs) that bind to the GDP-bound form and inhibit GDP/GTP exchange (maintaining the off-state) [134]. It was originally identified for its role in promoting phagocytosis through NADPH oxidase activation [135] and growth factor-induced membrane ruffling via actin polymerization [136].

Rac1 has been shown to participate in POS phagocytosis. Mao and colleagues demonstrated POS binding to αvβ5 integrin via MFG-E8 triggers two parallel signaling cascades in RPE cells: the Rac1/F-actin pathway and the FAK/MerTK pathway [66]. During phagocytosis, RPE cells exhibit both activation and subcellular redistribution of Rac1. While wild-type mice show elevated Rac1 activity during diurnal POS uptake, this response is absent in αvβ5 integrin mutants [66]. Pharmacological inhibition of Rac1 blocks F-actin recruitment to phagocytic sites in RPE cells, and its activation depends specifically on αvβ5 integrin and MFG-E8, but not on the FAK/MerTK pathway, with neither pathway affecting the other [66]. Further studies reveal that semaphorin 4D (sema4D) suppresses POS internalization in RPE cells by reducing Rac1 activity, while sema4D knockout mice display enhanced POS uptake at light onset [137]. Beyond phagocytosis, Rac1 signaling contributes to various RPE cellular functions, including cell migration [138], cytoskeletal rearrangement [139], and regulation of RPE maturation state [140].

### 3.6 LC3

The RPE clears its daily burden of POS through LC3-associated phagocytosis (LAP), a non-canonical autophagy pathway [31, 73]. LC3, a ubiquitously expressed soluble protein in mammalian cells, exhibits a molecular mass of approximately 17 kDa [141]. The human RPE expresses three distinct LC3 homologs (LC3A, LC3B, and LC3C), among which LC3B is expressed at markedly higher levels than LC3A and LC3C. The mouse RPE expresses both LC3A and LC3B isoforms, and genetic ablation of LC3B leads to decreased RPE POS phagosome clearance, abnormal accumulation of phagosomes, and contributes to AMD-like pathogenesis [142, 143]. Western blot detection of endogenous LC3 typically shows two distinct bands representing the unconjugated LC3-I and phosphatidyl-ethanolamine-conjugated LC3-II isoforms [144]. The lipidated form of LC3 (LC3-II) is essential for LAP through its selective association with phagosomal membranes, facilitating the targeted lysosomal clearance of engulfed material. Importantly, the ratio of LC3-II/-I is a well-characterized molecular signature for evaluating both conventional autophagy and LAP processes [141, 144].

Research has revealed that RPE cells achieve maximal conversion of LC3 to its lipidated LC3-II form at 7:00 AM, precisely one hour following light onset, which corresponds to the peak period of POS disc shedding [145]. Evidence suggests that melanoregulin may additionally coordinate the specific association between phagosomes and LC3 during POS disk processing [73]. As a master regulator of oxidative stress responses, Nrf2 (nuclear factor erythroid 2-related factor 2) activation enhances the maturation of POS-containing phagolysosomes and drives LC3-mediated phagocytic clearance via an AMPKα1-dependent mechanism in the RPE [146]. The initiation of autophagy critically depends on RB1 inducible coiled-coil 1 (RB1CC1), which acts as a key upstream regulator. Genetic ablation of RB1CC1 also markedly reduces LC3-I to LC3-II conversion and completely abolishes POS-stimulated autophagic activity in ARPE-19 cells [147]. Dysfunctional LAP mechanisms have been identified as pivotal drivers of RPE cell degeneration during AMD pathogenesis [32, 75, 76]. Retinal samples from age-matched individuals revealed progressive LC3 depletion during AMD progression, with early-stage cases showing moderate reduction, while late-stage specimens displayed significantly decreased protein expression relative to both non-AMD groups [148]. In addition, reduced LC3 intensity and phosphatidyl-ethanolamine-mediated LAP dysfunction contribute to defective phagolysosome activity in RPE cells, mediating Stargardt disease pathogenesis [149].

LAP and autophagy represent distinct yet interconnected pathways that share common molecular mediators and compete for intracellular resources, indicating their coordinated regulation under both physiological and stress conditions [150]. During peak periods of POS clearance in the morning, when LAP activity is maximally elevated, the cells exhibit upregulated Rubicon expression. This phagocytic activation concurrently stimulates epidermal growth factor receptor (EGFR) signaling, which triggers mechanistic target of rapamycin (MTOR) activation, along with subsequent SQSTM1/p62 accumulation and inhibitory phosphorylation of Beclin 1 at specific residues, collectively suppressing autophagy [17]. Notably, starvation can enhance autophagy while impairing POS degradation in RPE cells [17, 31, 151], demonstrating the mutually antagonistic relationship between these two degradation pathways.

## 4. Conclusions

Mounting evidence indicates that dysfunction in RPE phagocytosis leads to the accumulation of uncleared cellular debris, induces photoreceptor cell damage, disrupts retinal homeostasis, exacerbates tissue injury, and accelerates retinal degeneration. The limited understanding of the molecular mechanisms governing POS phagocytosis in RPE cells has hindered the development of therapies that modulate RPE phagocytic activity and prevent associated tissue degenerative damage. Novel research tools developed in recent years have enabled the systematic identification of phagocytic ligands, even in the absence of receptor information, and allowed for reliable quantification of their functional activity, thereby unraveling the molecular intricacies of RPE phagocytosis and dysfunction. However, there has been little investigation into potential therapeutic targets in RPE phagocytosis, such as MERTK and avβ5, and research on RPE phagocytic function has predominantly focused on cellular and animal studies, with a dearth of corresponding clinical data. Therefore, further research should prioritize the identification of various therapeutic targets and the characterization of their mechanisms of action to develop safe and efficacious treatment regimens. Moreover, the current understanding of the mechanisms and significance of POS phagocytosis is constrained by the absence of methods for imaging live-cell phagocytosis *in vivo*, which are essential to quantify the contribution of POS phagocytosis to both physiological health and disease pathogenesis. Addressing these issues can facilitate a more accurate evaluation of the true extent and significance of photoreceptor cell damage and retinal degeneration caused by RPE phagocytosis. Therapies targeting the RPE phagocytic pathway could potentially be developed to optimize phagocytic efficiency, restore retinal homeostasis, and prevent or delay the progression of retinal degeneration.
